# Prognostic significance of TRAIL death receptors in Middle Eastern colorectal carcinomas and their correlation to oncogenic KRAS alterations

**DOI:** 10.1186/1476-4598-9-203

**Published:** 2010-07-30

**Authors:** Prashant Bavi, Sarita E Prabhakaran, Jehad Abubaker, Zeeshan Qadri, Thara George, Nasser Al-Sanea, Alaa Abduljabbar, Luai H Ashari, Samar Alhomoud, Fouad Al-Dayel, Azhar R Hussain, Shahab Uddin, Khawla S Al-Kuraya

**Affiliations:** 1 Department of Human Cancer Genomic Research, MBC 98-16,Research Centre at KFNCCC, King Faisal Specialist Hospital and Research Centre,PO Box 3354, Riyadh 11211,Kingdom of Saudi Arabia; 2 Department of Pathology, King Faisal Specialist Hospital and Research Centre,PO Box 3354, Riyadh 11211,Kingdom of Saudi Arabia; 3 Department of Colorectal Surgery, King Faisal Specialist Hospital and Research Centre,PO Box 3354, Riyadh 11211,Kingdom of Saudi Arabia

## Abstract

**Background:**

Tumour necrosis factor-related apoptosis-inducing ligand (TRAIL) is a member of the tumour necrosis factor cytokine family that induces apoptosis upon binding to its death domain containing receptors, TRAIL receptor 1 (DR4) and TRAIL receptor 2 (DR5). Expression of TRAIL receptors is higher in colorectal carcinoma (CRC) as compared to normal colorectal mucosa and targeted therapy with TRAIL leads to preferential killing of tumor cells sparing normal cells.

**Methods:**

We investigated the expression of TRAIL and its receptors in a tissue microarray cohort of 448 Middle Eastern CRC. We also studied the correlation between TRAIL receptors and various clinico-pathological features including key molecular alterations and overall survival.

**Results:**

CRC subset with TRAIL-R1 expression was associated with a less aggressive phenotype characterized by early stage (p = 0.0251) and a histology subtype of adenocarcinomas (p = 0.0355). Similarly CRC subset with TRAIL-R2 expression was associated with a well-differentiated tumors (p < 0.0001), histology subtype of adenocarcinomas (p = 0.0010) and tumors in left colon (p = 0.0009). Over expression of pro apoptotic markers: p27^KIP1 ^and KRAS4A isoforms was significantly higher in CRC subset with TRAIL-R1 and TRAIL-R2 expression; TRAIL-R1 expression was also associated with cleaved caspase-3(p = 0.0011). Interestingly, TRAIL-R2 expression was associated with a microsatellite stable (MS--S/L) phenotype (p = 0.0003) and with absence of KRAS mutations (p = 0.0481).

**Conclusion:**

TRAIL-R1 expression was an independent prognostic marker for better survival in all CRC samples and even in the CRC group that received adjuvant therapy. The biological effects of TRAIL in CRC models, its enhancement of chemosensitivity towards standard chemotherapeutic agents and the effect of endogenous TRAIL receptor levels on survival make TRAIL an extremely attractive therapeutic target.

## Introduction

Colorectal Cancer (CRC) is a major cause of mortality and morbidity worldwide. In Saudi Arabia, the incidence of CRC is increasing. According to the latest statistics, CRC is the second most common cancer among Saudi males and the third most common among Saudi females [1]. Currently available therapeutic approaches for CRC are less effective, and thus the prognosis is poor. Despite a growing number of publications about biomarkers that give information on disease outcome, the best prognostic factors are still simple clinical parameters like number of lymph nodal metastasis, presence of distant metastasis, tumour grade and AJCC stage. Prognostic biomarkers might especially be useful for hypothesis testing for their relevance as predictive markers, as targets for therapy and for the selection of patients for adjuvant treatment [2].

Apoptosis, or programmed cell death, is a major control mechanism by which cells die if DNA damage is not repaired [3]. Apoptosis is an essential biochemical pathway for normal tissue homeostasis, cellular differentiation, and development. Derangements of normal apoptotic mechanisms provide a growth advantage to cancer cells [4]. The understanding of apoptosis has provided the basis for novel targeted therapies that can induce death in cancer cells or sensitize them to established cytotoxic agents and radiation therapy [5]. In addition, as apoptosis usually does not elicit host inflammatory or immune response, this type of cell death is the preferred way of cancer cell killing by various treatments. Accordingly, selectively inducing apoptosis in tumour cells is gaining recognition as a promising therapeutic approach for many cancers [6]. Tumour necrosis factor-related apoptosis-inducing ligand (TRAIL or Apo2 ligand) is a member of the tumour necrosis factor (TNF) cytokine family that induces apoptosis upon binding to its death domain containing receptors, TRAIL receptor 1 (death receptor 4, DR4) and TRAIL receptor 2 (death receptor 5, DR5) [7]. The TRAIL receptors, TRAIL-R1 and TRAIL-R2, are highly expressed in many cancer cells including CRC [8-10]. A further three TRAIL receptors exist, which are unable to induce apoptosis and act as decoys. Decoy receptors 1 (DcR1) and 2 (DcR2), similar to TRAIL-R1 and TRAIL-R2, are expressed on the cell surface. Thus, overexpression of either DcR1 or DcR2 confers protection against TRAIL-induced apoptosis [11,12]. The fifth TRAIL receptor is osteoprotegerin (OPG), a secreted, low affinity receptor for TRAIL [11,12]. Binding of TRAIL to TRAIL-R1 and TRAIL-R2 induces trimerization of TRAIL-R1 and TRAIL-R2 [13]. The trimerized TRAIL-R1 and TRAIL-R2 bind to FADD, which recruits caspase 8 and initiates a proteolysis cascade that eventually leads to cell death by apoptosis. Many cancer cells are resistant to death receptor induced apoptosis [4]. The mechanisms of resistance include the presence of decoy receptors for TRAIL [12], the loss of TRAIL receptor expression [14], the overexpression of inhibitory proteins in signal transduction pathways such as FLICE-inhibitory protein [14], and the mutation of TRAIL-R2 gene [15-18].

Oncogenic mutations such as ras may enhance expression of TRAIL receptors; potentially sensitizing these tumors to TRAIL based therapies [19-21]. Constitutively activated Ras increases the tumorigenic potential of cells because it causes deregulation of important intracellular signaling pathways [22]. Activated RAS mediates its biological activity through interaction with various downstream effector targets, thus activating pathways like MEK, PI3K, and Rho GTPases [22,23]. RAS regulates a RAF-MEK-ERK1/2 kinase cascade and this pathway is found to be active in human colon adenocarcinomas cells [24] as well as in human colorectal tumors [25]. Drosopoulos *et al. *[21] have shown transformation of the colon cell line Caco-2 by ras oncogenes sensitizes these cells to TRAIL induced apoptosis by causing specific MEK-dependent up-regulation of TRAIL-R1 and TRAIL-R2. Nesterov A et al. [20] have demonstrated that normal cells are sensitized to TRAIL when TRAIL-R2 is up regulated by overexpression of c-myc or oncogenic ras mutants. Thus, RAS-MEK-ERK1/2 signaling pathway can sensitize cells to TRAIL-induced apoptosis by up-regulating TRAIL-R1, TRAIL-R2 and TRAIL-based therapeutic strategies using TRAIL agonists could be used in cases of human colon cancers bearing RAS mutations. Therefore, we also sought to explore the potential link between expression of TRAIL and its receptors with KRAS alterations in CRC.

The aims of the present study were: (*a*) to determine the TRAIL/TRAIL receptor expression pattern in normal and neoplastic colon epithelium; (*b*) to correlate immunohistochemical expression patterns with KRAS alterations, microsatellite instability and pro apoptotic markers; *(c) *to correlate immunohistochemical expression patterns with overall survival.

## Results

### Expression of TRAIL and its receptors TRAIL-R1 and TRAIL-R2

**Figure 1:**
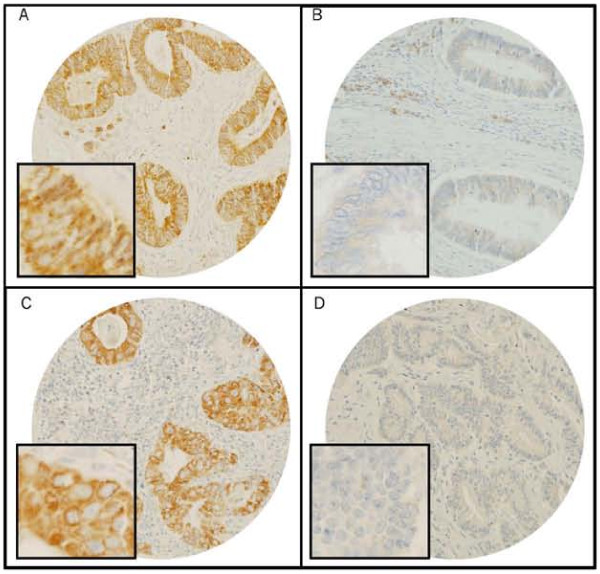
**Tissue microarray-based immunohistochemical analysis of TRAIL receptors TRAIL-R1 and TRAIL-R2 in CRC patients**. CRC array spots showing overexpression of TRAIL-R1 (A) and TRAIL-R2 (C). In contrast, other CRC tissue array spots showing low expression of TRAIL-R1 (B) and TRAIL-R2 (D). 20 ×/0.70 objective on an Olympus BX 51 microscope (Olympus America Inc, Center Valley, PA, USA), with the inset showing a 40 ×/0.85 aperture magnified view of the same

**Figure 2:**
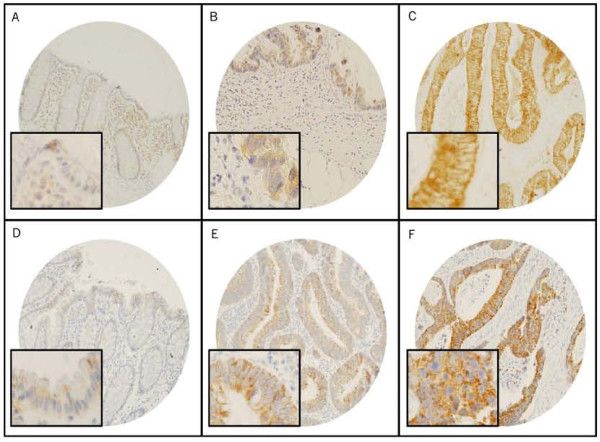
**Tissue microarray-based immunohistochemical analyses of TRAIL receptors TRAIL-R1 and TRAIL-R2 in CRC carcinogenesis**. A progressive increase on TRAIL-R1 expression was seen from normal colorectal mucosa (A) to adenoma (B) to carcinoma(C). Similarly, a progressive increase on TRAIL-R2 expression was seen from normal colorectal mucosa (D) to adenoma (E) to carcinoma (F).

**Figure 3:**
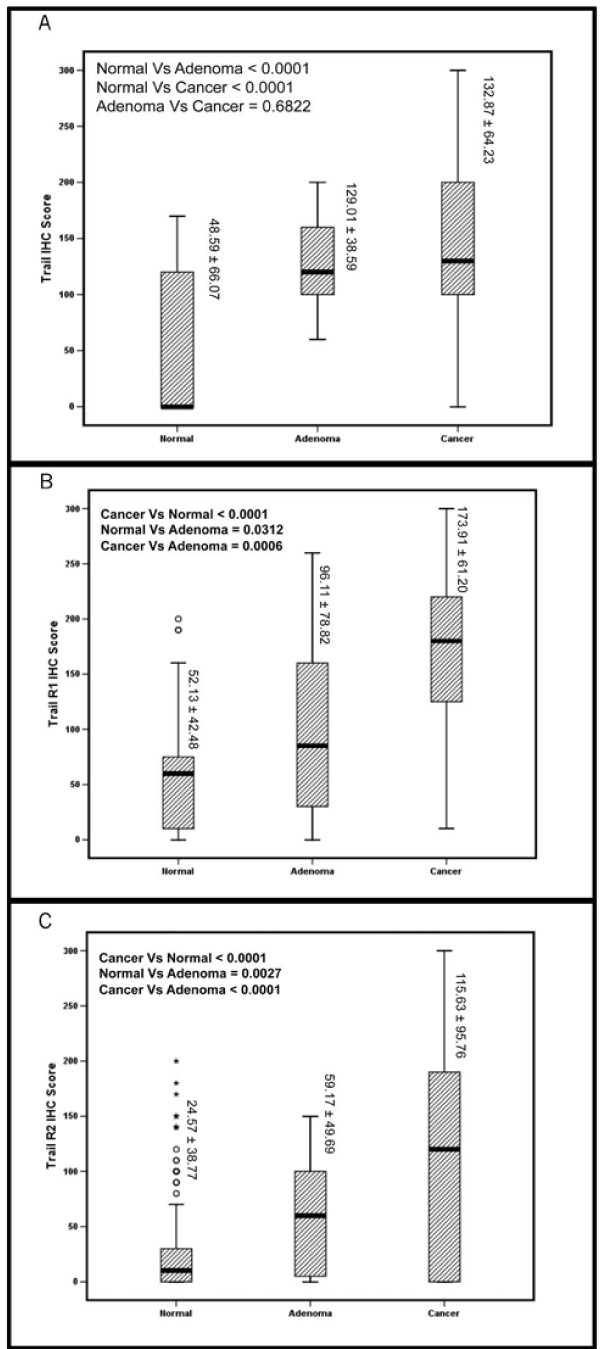
**Box plot of TRAIL, TRAIL-R1 and TRAILR2 expression in cancer, adenoma, and normal samples of colorectal patients**. **(A) **Using Student's t-test, the mean ± SD of TRAIL expression in normal colorectal mucosa (48.59 ± 66.07), adenoma (129.01 ± 38.59) and cancer (132.87 ± 64.23) and showed a significant association with cancer versus normal (p < 0.0001) and adenoma versus normal (p < 0.0001). **(B) **Using Student's t-test, the mean ± SD of TRAIL-R1 expression in normal colorectal mucosa (52.13 ± 42.48), adenoma (96.11 ± 78.82), cancer (173.91 ± 61.20) showed a significant association with cancer versus normal (p < 0.0001), adenoma versus normal(p = 0.0312) and adenoma versus cancer(p = 0.0006). **(C) **Using Student's t-test, the mean ± SD of TRAIL-R2 expression in normal colorectal mucosa (24.57 ± 38.77), adenoma (59.17 ± 49.69) and cancer (115.63 ± 95.76) showed a significant association with cancer versus normal (p < 0.0001), adenoma versus normal(p = 0.0027) and adenoma versus cancer(p < 0.0001).

Incidence of TRAIL-R1, TRAIL-R2 and TRAIL ligand expression in CRC was 85.5% (331/387), 59.4(217/365) and 31.5% (127/403) respectively [Figure 1]. These incidences are within the wide ranges reported earlier - TRAIL: 37.5% to 83%, TRAIL-R1:58.1% to 100.0% and TRAIL-R2: 40.3% to 100% [26-31]. Incidence of non-interpretable tumor spots for TRAIL, TRAIL-R1 and TRAIL R2 ranged from 10 to 18%. Tumor spots were deemed not interpretable if they had insufficient tumor cells, loss of tissue in the spot, or an abundance of necrotic tissue. Expression of TRAIL and its receptors was also evaluated in colorectal adenomas and adjacent colorectal mucosa [Figure 2]. Both TRAIL-R1 and TRAIL-R2 expression was significantly higher in both colorectal adenomas [TRAIL-R1 96.11 ± 78.82(p = 0.0312); TRAIL-R2 59.17 ± 49.69(p = 0.0027)] and carcinoma [TRAIL-R1 173.91 ± 61.20(P < 0.00001); TRAIL-R2 115.63 ± 95.76(p = < 0.0001)] as compared to normal colorectal mucosa (TRAIL-R1 52.13 ± 42.48; TRAIL-R2 24.57 ± 38.77). In addition, there was a significant difference in expression of both TRAIL-R1 (p = 0.0006) and TRAIL-R2 (p = < 0.0001) between colorectal adenomas and carcinoma [Figure 3B and 3C]. Similarly, TRAIL expression was significantly higher in carcinoma (132.87 ± 64.23; p < 0.0001) and adenomas (129.01 ± 38.59; p < 0.0001) as compared to normal colorectal mucosa (48.59 ± 66.07). However, there was no difference in TRAIL expression between adenomas and carcinomas (p = 0.6822; Figure 3A). Thus the TRAIL system may play a key role in colorectal carcinogenesis.

### Association of TRAIL, TRAIL-R1 and TRAIL-R2 with clinico-pathological parameters

TRAIL-R1 was associated with histology subtype of adenocarcinomas (p = 0.0355), early AJCC stage (p = 0.0251) and a trend of higher expression was noted with well-differentiated tumors (p = 0.0887). No association was seen with age, gender and tumor site (Table 1). Similarly, TRAIL-R2 was associated with histology subtype of adenocarcinomas (p = 0.0010, tumors in the left colon (p = 0.0009) and a significantly higher expression was noted with well-differentiated tumors (p < 0.0001). No associations were seen with age, gender and tumor stage (Table 2). TRAIL ligand expression was not associated with any of the clinico-pathological parameters (see Additional File 1 Table S1).

**Table 1:** Clinico-pathological characteristics and TRAIL-R1 expression of patients with colorectal carcinoma

			High TRAIL-R1		Low TRAIL-R1		p value
				
	N	%	N	%	N	%	
**Total Number of Cases**	387		331	85.5	56	14.5	

**Age**							
< = 50 years	126	32.6	110	87.3	16	12.7	0.4870
> 50 years	261	67.4	221	84.7	40	15.3	

**Gender**							
Male	193	49.9	167	86.5	26	13.5	0.5773
Female	194	50.1	164	84.5	30	15.5	

**Tumour Site**							
Left colon	324	83.7	276	85.2	48	14.8	0.6576
Right colon	63	16.3	55	87.3	8	12.7	

**Histological Type**							
Adenocarcinoma	334	86.3	291	87.1	43	12.9	0.0355
Mucinous Carcinoma	53	13.7	40	75.5	13	24.5	

**Tumour Stage**							
I	55	15.0	53	96.4	2	3.6	0.0251
II	126	34.2	105	83.3	21	16.7	
III	141	38.3	121	85.8	20	14.2	
IV	46	12.5	36	78.3	10	21.7	

**Differentiation**							
Well	33	8.5	30	90.9	3	9.1	0.0887
Moderate	284	73.4	247	87.0	37	13.0	
Poor	70	18.1	54	77.1	16	22.9	

**KRAS Mutation**							
Present	75	28.3	67	89.3	8	10.7	0.7407
Absent	190	71.7	167	87.9	23	12.1	

**K-RAS 2A**							
High expression	155	46.7	150	96.8	5	3.2	< 0.0001
Low expression	177	53.3	142	80.2	35	19.8	

**Cleaved-Caspase 3**							
High expression	172	49.9	160	93.0	12	7.0	0.0011
Low expression	173	50.1	141	81.5	32	18.5	

**P27 (Nuc)**							
High expression	139	38.4	130	93.5	9	6.5	0.0024
Low expression	223	61.6	185	83.0	38	17.0	

**MSI-Molecular**							
MSI-H	67	18.7	56	83.6	11	16.4	0.4731
MSI-S/L	292	81.3	254	87.0	38	13.0	

**Overall Survival**							
5 Years				68.1		53.9	0.0124

**Table 2:** Clinico-pathological characteristics and TRAIL-R2 expression of patients with colorectal carcinoma

			High TRAIL-R2		Low TRAIL-R2		p value
				
	N	%	N	%	N	%	
**Total Number of Cases**	365		217	59.4	148	40.6	

**Age**							
< = 50 years	121	33.1	71	58.7	50	41.3	0.8320
> 50 years	244	66.9	146	59.8	98	40.2	

**Gender**							
Male	172	47.1	101	58.7	71	41.3	0.7883
Female	193	52.9	116	60.1	77	39.9	

**Tumour Site**							
Left colon	309	84.7	195	63.1	114	36.9	0.0009
Right colon	56	15.3	22	39.3	34	60.7	

**Histological Type**							
Adenocarcinoma	323	88.5	202	62.5	121	37.5	0.0010
Mucinous Carcinoma	42	11.5	15	35.7	27	64.3	

**Tumour Stage**							
I	46	13.3	33	71.7	13	28.3	0.2842
II	117	33.9	65	55.6	52	44.4	
III	137	39.7	84	61.3	53	38.7	
IV	45	13.0	28	62.2	17	37.8	

**Differentiation**							
Well	28	7.7	22	78.6	6	21.4	**< 0.0001**
Moderate	276	75.6	176	63.8	100	36.2	
Poor	61	16.7	19	31.1	42	68.9	

**KRAS Mutation**							
Present	67	29.9	41	61.2	26	38.8	0.0481
Absent	157	70.1	117	74.5	40	25.5	

**K-RAS 2A (cyto)**							
High expression	160	46.8	128	80.0	32	20.0	< 0.0001
Low expression	182	53.2	82	45.1	100	54.9	

**Cleaved-Caspase 3**							
High expression	164	49.8	109	66.5	55	33.5	0.1209
Low expression	165	50.2	96	58.2	69	41.8	

**P27 (Nuc)**							
High expression	135	40.2	102	75.6	33	24.4	< 0.0001
Low expression	201	59.8	105	52.2	96	47.8	

**MSI-Molecular**							
MSI-H	66	19.8	28	42.4	38	57.6	0.0003
MSI-S/L	267	80.2	178	66.7	89	33.3	

**Overall Survival**							
5 Years				67.3		57.6	0.0211

### Association of TRAIL, TRAIL-R1 and TRAIL-R2 with KRAS mutations and KRAS splice variants KRAS4A and KRAS4B

**Figure 4:**
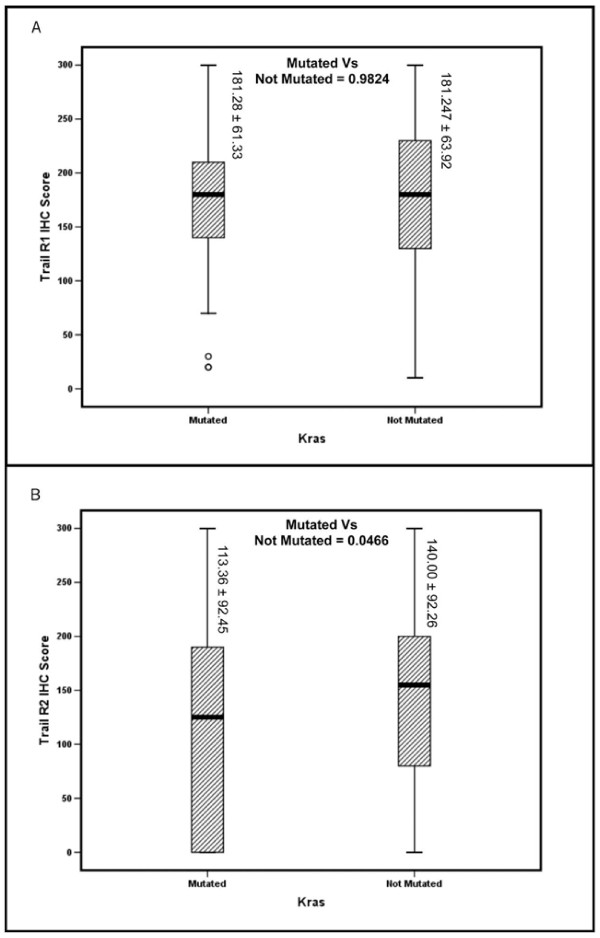
**Box plot analysis of TRAIL-R1 and TRAIL-R2 expression in KRAS mutated versus non-mutated colorectal patients**. **(A) **Using Student's t-test, the mean ± SD of TRAIL-R1 expression in KRAS mutated (181.28 ± 61.33)and non mutated (181.25 ± 63.92).However, this difference in expression was not statistically significant (p = 0.9824). **(B **Using Student's t-test, the mean ± SD of TRAIL-R2 expression in KRAS mutated (113.36 ± 92.45) and not mutated (140.00 ± 92.26).There was statistically significant difference in expression (p = 0.0466).

TRAIL-R2 expression was significantly higher in the CRC subset lacking KRAS mutations as compared to CRC with KRAS mutations (p = 0.0481; Figure 4**). **Interestingly, both TRAIL-R1(p < 0.0001) and TRAIL-R2(p < 0.0001) showed a highly significant association with the pro-apoptotic KRAS4A isoform. However, TRAIL-R1 expression did not show any correlations with KRAS mutations and KRAS4B isoform (Table 1 &2). TRAIL expression did not show any associations with KRAS mutations or expression of KRAS splice variants (see Additional File 1 Table S1).

### Associations of TRAIL, TRAIL-R1 and TRAIL-R2 with microsatellite instability, cleaved caspase 3 and p27^kip1^

p27^kip1 ^expression was significantly associated with both TRAIL-R1 (p = 0.0024) and TRAIL-R2 (p < 0.0001; Table 1 &2). CRC with expression of TRAIL-R1 but not TRAIL-R2 or TRAIL also showed expression of cleaved caspase3 (p = 0.0011). Although TRAIL-R2 was associated with a phenotype of microsatellite stable (MSI-S/L) tumors (p = 0.0003), no associations were seen between TRAIL-R1 or TRAIL and microsatellite instability status.

### Overall survival in all patients, selected stage subgroups and combination groups of TRAIL receptors

**Figure 5:**
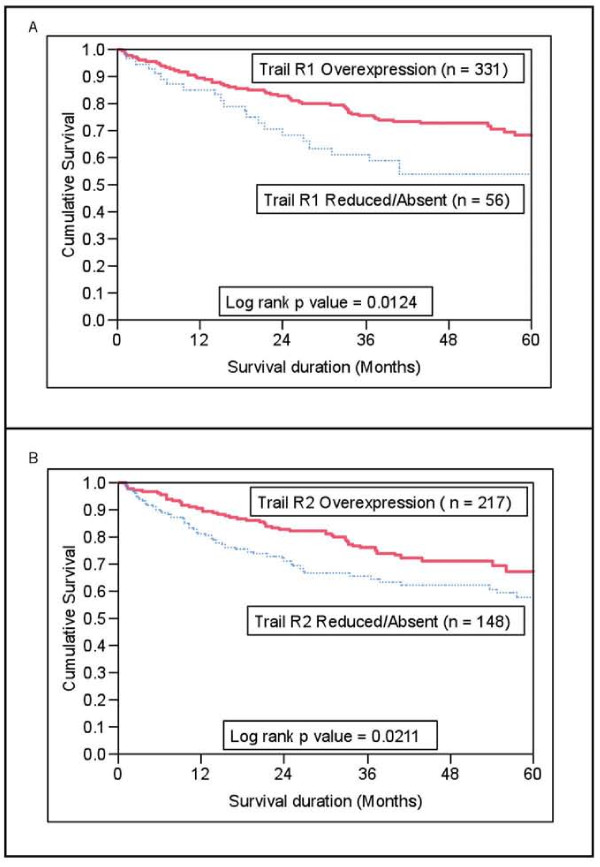
**(A) Kaplan Meier survival analysis in CRC patients with over expression of Trail R1 had a better overall survival of 68.1% at 5 years as compared to 53.9% with reduced TRAIL-R1 expression (p = 0.0124)**. **(B) **CRC patients Trail R2 also showed a better overall survival as of 67.3% compared to 57.6% with reduced TRAIL-R2 expression ( p = 0.0211).

CRC with low TRAIL-R1 expression also showed a poor 5 year overall survival of 53.9% as compared to 68.1% with high TRAIL-R1 expression (p = 0.0124; Figure 5A). Similarly, CRC with low TRAIL-R2 expression also showed a poor 5 year overall survival of 57.6% as compared to 67.3% with high TRAIL-R2 expression (p = 0.0211; Figure 5B). TRAIL expression did not show any prognostic significance (p = 0.2901). To exclude that the observed prognostic difference were caused by classical prognostic factors of CRC, we performed a multivariate analysis (Cox proportional hazards) with histological subtype, tumor grade, tumor stage, age, gender and microsatellite instability status as variables (Table 3). In the multivariate analysis, only TRAIL-R1 expression retained its significance. The relative risk was 1.84 (for low TRAIL-R1 expression (95% CI 1.10 - 3.02; p = 0.0273) and 6.56 for high stage group III-IV (95% CI 3.67- 12.78; p = < 0.0001). Thus, TRAIL-R1 was an independent prognostic marker in Middle Eastern Colorectal Carcinoma. To exclude that TRAIL-R1 is not a readout of KRAS-4A or p27 we reanalyzed our data and did a Cox proportional hazards model where we included age, gender, Stage, Grade, KRAS-4A, p27 and TRAIL-R1 expression (see Additional File 1 Table S2). In a Cox proportional Hazards model, the independent prognostic significance of TRAIL-R1 was weakened (p = 0.0883). However, AJCC stage, p27 and KRAS4A still remained independent prognostic markers.

**Table 3:** TRAIL-R1 expression: Cox regression analysis for overall survival of patients with colorectal carcinoma

	UNIVARIATE		MULTIVARIATE	
	
Clinical Parameters	Risk Ratio (95% CI)	p value	Risk Ratio (95% CI)	p value
**Age: **Above = 50	1.17 (0.80-1.74)	0.4306	1.40 (0.86 - 2.33)	0.1755
**Sex: **Male	1.10 (0.76-1.59)	0.6084	1.18 (0.75 - 1.87)	0.4589
**Stage: **III-IV	7.26 (4.34-13.03)	< 0.0001	6.56 (3.67 - 12.78)	< 0.0001
**Grade: **Poorly differentiated	1.41 (0.90-2.14)	0.1307	3.61 (1.72 - 6.80)	0.0014
**MSI status: **MSI-L/S	2.04 (1.14 - 4.05)	0.0149	1.90 (0.95 - 4.37)	0.0713
**Histology: **Adenocarcinoma	1.14 (0.67 - 2.10)	0.6499	2.28 (0.93 - 5.43)	0.0695
**TRAIL-R1: **Low expression	1.18 (1.11 - 2.83)	0.0196	1.84 (1.10 - 3.02)	0.0273

CI = confidence interval

Although TRAIL-R1 expression was significantly more in early stage tumors, a vast majority of Stage III & IV tumors (82%) also showed TRAIL-R1 expression. Both TRAIL-R1 (p = 0.0060) and TRAIL-R2 (p = 0.0263) were associated with better outcome only in the advanced Stage group (III and IV; see Additional File 2). When stage II and III were taken together only TRAIL-R2 expression was associated with better overall survival (p = 0.0088); TRAIL-R1 expression was not significant (p = 0.2508; see Additional File 3). Co-expression of TRAIL-R1 and TRAIL-R2 was seen in 56.85% of the CRC( 191/336) and was associated with a good survival (p = 0.0107; see Additional File 4) which remained significant in multivariate analysis (Cox proportional hazards) with TRAIL-R1/R2 co-expression, tumor grade, tumor stage, age and gender as variables (see Additional File 1 Table S3).

### TRAIL death receptors and response to adjuvant therapy

The availability of 220 CRC from affected individuals who had undergone adjuvant therapy: chemotherapy and/or radiotherapy, allowed us to investigate the possible impact of TRAIL-R1 on response to adjuvant therapy. For this analysis, we first stratified the individuals into two groups: (i) CRC patient who have received adjuvant therapy (n = 220), and (ii) CRC patient who have been treated by surgical resection only and have not received adjuvant therapy (n = 90). There was a statistically significant difference in survival between individuals with tumors with TRAIL-R1 overexpression versus those with reduced expression (p = 0.0033; see Additional File 5). To exclude that the observed prognostic difference was caused by classical prognostic factors of CRC we performed a multivariate analysis (Cox proportional hazards) with TRAIL-R1 expression, tumor grade, tumor stage, age and gender as variables (see Additional File 1 Table S4). We found that the prognostic value of TRAIL-R1 expression in adjuvant treated individuals was independent of these factors. Similarly, TRAIL-R2 expression was also associated with trend towards better outcome in the adjuvant treated CRC subgroup (p = 0.0998) but no association with outcome was seen in the group which did not receive adjuvant therapy.

## Discussion

We conducted this study to examine the relations of TRAIL and it receptors: TRAIL-R1 and TRAIL-R2 with clinical, pathologic, molecular characteristics and patient survival in Saudi colorectal cancers. Expression of TRAIL-R1 or TRAIL-R2 was associated with a less aggressive phenotype characterized by an early AJCC stage and well-differentiated tumors. TRAIL-R2 expression was associated with microsatellite stable phenotype and with absence of KRAS mutations. TRAIL-R1 but not TRAIL-R2 was an independent prognostic marker for better survival.

Using immunohistochemistry, we have studied the expression of TRAIL and its receptors in Saudi CRC; incidence of TRAIL R1, TRAIL-R2 and TRAIL expression was 85.5%, 59.4% and 31.5% respectively. In agreement with earlier studies, we have also observed a progressive increase in expression of TRAIL and its receptors: TRAIL-R1 and TRAIL-R2 in colorectal carcinoma and noted a strong association of TRAIL -R1 or TRAIL-R2 expression with differentiation and an early stage. The prognostic implication of TRAIL receptor expression is the subject of intensive investigation as malignant cells are more sensitive to TRAIL- induced apoptosis than their benign counterparts are and this potentially affects the future management of patients [32-34]. Furthermore, our data indicates that high TRAIL-R1 expression was an independent prognostic marker for better survival in Saudi CRC patients. TRAIL-R2 was also associated significantly with better outcome but failed to remain significant in multivariate analysis. TRAIL-R1 expression was also associated with better outcome in the following subgroups: Stage III and IV (p = 0.0060) and CRC subgroup who received adjuvant therapy(p = 0.0033). To elucidate the role of TRAIL expression further analysis was done in the following subgroup: CRC subgroup with high co-expression of TRAIL and TRAIL-R1 and CRC subgroup with high co-expression of TRAIL and TRAIL-R2. Both these combination groups were not associated with outcome (data not shown). Thus, TRAIL ligand co-expression with TRAIL receptors does not influence the outcome.

These findings are in agreement with earlier studies by Starter et al [26] where TRAIL-R1 expression was associated with a better disease free survival in a cohort of 129 Stage II and III CRC [26]. Granci et al. [28] studied the TRAIL receptors TRAIL-R 1, -2, -3 and -4 expression by immunohistochemistry in metastatic stage IV CRC and found that concomitant low/mediumTRAIL-R1 and high TRAIL-R3 expression in primary CRC is significantly associated with a poor response to 5-FU-based first-line chemotherapy and with a shorter progression-free survival. Surprisingly, high TRAIL R1 was associated with worse disease free survival and overall survival in 376 CRC patients with Stage III [30]. Ullenhag et al. [29] analyzed FLICE inhibitory protein (c-FLIP) and TRAIL receptors(TRAIL-R1 and R-2) in 476 CRC of all Stage groups(I to IV): Overexpression of FLIP_L_, (the long form of FLICE inhibitory protein) but not TRAIL-R1 or TRAIL-R2, was an independent prognostic factor for shorter disease free survival. In an attempt to explain these conflicting results of TRAIL and its pro-apoptotic receptors in CRC, we offer the following explanations: a) differences and heterogeneity in samples studied: sample size, ethnic differences, different Stage groups, tumor site- colon or rectal tumors, type of treatment- surgery and/or chemo/radiotherapy; b) differences in scoring system could be another important reason for this difference. The varied effects of TRAIL signaling could be also attributed to the following factors: TRAIL resistance due to presence of decoy receptors [11], number, type and functionality of TRAIL receptors [7,12] and intracellular anti apoptotic molecules like c-FLIP [35], IAP [36], Mcl-1 [37] and bcl2 [38].

Although TRAIL-R1 lost its statistical significance when included as a prognostic marker in multivariate analysis with p27 and KRAS4A (see Additional File 1 Table S2), this does not argue against the biological role of TRAIL-R1 in CRC as much as it reflects that p27 and KRAS4A are a more powerful predictor of clinical outcome of CRC than TRAIL-R1 expression. We can hypothesize that the TRAIL-R1 functions most effectively in the cells which show co-expression of p27^kip1 ^in concordance with an earlier study [39]. Despite some studies that show a role of Ras signaling pathway in modulating the TRAIL system, studies on the KRAS isoforms - KRAS4A and KRAS 4B are lacking. Alternate approaches to modulate the expression of KRAS isoforms, a greater understanding of the role(s) that each oncoprotein plays in malignant transformation, including the signal transduction pathways affected, is crucial in the development of therapeutic approaches in cancer treatment, which include the use of drugs that target isoform-specific post-translational modifications [40] and of antisense oligonucleotides to modulate alternative splicing [41].

Oncogenic mutations such as ras may enhance expression of TRAIL receptors; potentially sensitizing these tumors to TRAIL based therapies [19-21]. TRAIL-based therapeutic strategies using TRAIL agonists could be used in cases of human colon cancers bearing RAS mutations. In a small cohort of 51 CRC, Oikonomou E *et al.*[42] have reported a much lower incidence of KRASG12/13 mutations(10%) and have concluded that there is clear correlation between these mutations(KRAS and BRAF) and upregulation of TRAIL-R1 and TRAIL-R2. Despite lack of statistical significance they have concluded that CRC with mutations in KRAS or BRAF gene had significantly upregulated both TRAIL death receptors. In our earlier study [43] KRAS gene mutations were seen in 80/285 CRC (28.1%) and were an independent prognostic marker for poor survival. Interestingly we have observed a significantly higher expression of TRAIL-R2 (p = 0.0481) in CRC subgroup lacking KRAS mutations(75.5%) as compared to the CRC subgroup with KRAS mutations(61.1%). In view of the recent findings of KRAS mutations and PIK3CA mutations contributing to resistance to EGFR inhibitors like Cetuximab,[44,45] a better understanding of the TRAIL system with context to KRAS mutations might be useful. The KRAS gene has two alternative fourth exon variants that result from differential splicing and activating mutations affect both isoforms [46-48]. Studies in animals indicate that KRAS4A promotes apoptosis while KRAS4B inhibits it, and KRAS4B promotes differentiation [49,50]. In our study [43], KRAS 4A a pro apoptotic isoform, in particular was found to be an independent prognostic marker for better survival in all CRC patients. Even in the CRC subgroup lacking KRAS mutations KRAS4A was associated with better survival. Furthermore, we have observed a highly significant association of KRAS4A and both the TRAIL receptors: TRAIL-R1(p < 0.0001) and TRAIL-R2(p < 0.0001). Considering the tight linkage between TRAIL-R1 and KRAS4A future studies should be conducted to understand the association between these markers.

In summary, our study shows high TRAIL-R1 expression to be an independent prognostic marker for better survival in colorectal cancer. High TRAIL-R1 or TRAIL-R2 expression was associated with a less aggressive phenotype characterized by early AJCC stage, well-differentiated tumors, microsatellite stable cancers, absence of KRAS mutations and expression of pro apoptotic molecules: KRAS4A, p27^kip1 ^and cleaved caspase 3. Further work is needed to elucidate the biological significance of high TRAIL-R1 expression and better outcome, and to establish the association between TRAIL-R1 expression and response to therapy that targets this receptor. The biological effects of TRAIL in CRC models, its enhancement of chemosensitivity with standard chemotherapeutic agents and the effect of endogenous TRAIL receptor levels on survival make TRAIL an extremely attractive therapeutic target.

## Materials and methods

### Patient selection and tissue microarray construction

Four hundred forty eight patients with CRC diagnosed between 1990 and 2006 were selected from King Faisal Specialist Hospital and Research Centre. All CRC, 24 adenomas and 229 adjacent normal colorectal mucosa were analyzed in a tissue microarray format. Clinical and histopathological data were available for all these patients. Colorectal Unit, Department of Surgery, provided long-term follow-up data. From our cohort of 448 patients treatment details were available for 310 patients:220 patients received adjuvant therapy; 90 were treated by surgery alone and 138 patients were excluded as we could not retrieve treatment details. Patients with colon cancer underwent surgical colonic resection and those with rectal cancer underwent anterior resection or abdominoperineal resection. All node-positive colon cancers received 5-fluorouracil-based adjuvant chemotherapy. A vast majority of the rectal cancers received radiotherapy alone or chemoradiotherapy prior to surgery, followed by adjuvant chemotherapy after surgery. Fixation of tissues was done overnight with 10% neutral buffered formalin at the Pathology Laboratory of King Faisal Specialist Hospital and Research Centre, Riyadh. Tissue microarrays were constructed from formalin-fixed, paraffin-embedded colorectal carcinoma specimens as described previously [51]. One pathologist (PB) reviewed all tumors for grade and histological subtype. Institutional Review Board (IRB) of the King Faisal Specialist Hospital & Research Centre approved the study.

### Immunohistochemistry (IHC)

Tissue microarray slides were processed and stained manually. The streptavidin-biotin peroxidase technique with diaminobenzidine as chromogen was applied. For antigen retrieval, Dako Target Retrieval Solution was used at a pH of 6 for TRAIL-R1 and pH of 9 for TRAIL-R2 was used, and the slides were microwaved at 750W for 5 minutes and then at 250W for 30 minutes. Primary antibodies used, their dilutions, and incidences are listed in Additional File 1 Table S5. The specificity of these antibodies for TRAIL and its receptors has been previously assessed by immunohistochemistry [28,52], or by Western blot [53]. Endogenous peroxidase activity was quenched using 3% hydrogen peroxidase. Endogenous biotin was blocked and all slides were counterstained with hematoxylin, dehydrated, cleared, and cover slipped with premount. Only fresh cut slides were stained simultaneously to minimize the influence of slide ageing and maximize repeatability and reproducibility of the experiment. As controls, we used a tissue microarray control block comprising multiple cores from normal tissue from various sites, common epithelial cancers and colon cancer cell lines. Omission of the primary antibody also served as a negative control for TRAIL, TRAIL-R1 and -R2 staining.

### Immunohistochemistry Assessment

TRAIL-R1, TRAIL-R2 and TRAIL expression was categorized by doing an H score [54,55]. Each tissue microarray spot was assigned an intensity score from 0-3(I_0_, I_1-3_) and proportion of the tumor staining for that intensity was recorded as 5% increments from a range of 0-100(P_0_, P_1-3_). A final H score (range 0-300) was obtained by adding the sum of scores obtained for each intensity and proportion of area stained (H score = I_1X_P_1_+I_2_XP_2_+I_3_XP_3_). CRCs were grouped into two groups based on X-tile plots for TRAIL-R1: one with complete absence or reduced staining (H score = 0-110) and the other group showed over expression (H score > 110) depending on the H score. Similarly, X-tile plots were used to stratify the CRC cases into two groups for TRAIL-R2 and TRAIL. X-tile plots were constructed for assessment of biomarker and optimization of cut off points based on outcome as has been described earlier [56,57]. For cleaved caspase-3 expression, we used the antibody clone C5A**-1 **from Cell signalling technologies as described previously [43]. CRCs were grouped into two groups based on X-tile plots: one with complete absence or reduced staining (H score = 0-9 for low cleaved caspase3); and the other group showed over expression (H score >9 for high cleaved caspase 3). Grading of p27 nuclear protein staining was based on proportion or percentage of cell nuclei staining and was semi quantified as high or low. Nuclear protein expression of epithelial cells only was scored as high if 50% or more of the nuclei were stained or low if < 50% were stained as described previously [58]. This scoring criteria has been used earlier [59].

### Mutational analysis of the KRAS gene

KRAS mutations were done as described earlier [43]. Briefly the step-down cycling condition was used for the detection of exon 1 mutation of the KRAS gene. After 10 minutes denaturing at 95°C, the PCR was run with each temperature for 1 min at five step-down steps, for two cycles each. The denaturing temperature was 95°C and the extension temperature was 72°C for each step, with an annealing temperature of 66°C, 64°C, 62°C, 60°C, and 58°C from the first to the last step. The PCR was finally run at 95°C, 58°C, and 72°C, each for 1 min for 35 cycles, followed by an elongation at 72°C for 5 min. The PCR products were subsequently subjected to direct sequencing PCR with BigDye terminator V 3.0 cycle sequencing reagents (Applied Biosystems, Foster City, CA, USA). The samples were finally analysed on an ABI PRISM 3100xl Genetic Analyzer (Applied Biosystems).

### Microsatellite instability

Allelic imbalances were measured by performing microsatellite analysis on all matched normal and tumor tissue by PCR amplification as described previously [60]. A reference panel of five pairs of microsatellite primers, comprising two mononucleotide microsatellites (BAT25, BAT26) and three dinucleotide microsatellites (DS123, D5S346 and D17S250) were used to determine tumor MSI status. Multiplex PCR was performed in a total volume of 25 ml using 50 ng of genomic DNA, 2.5 ml 10 Taq buffer, 1.5 ml MgCl2 (25 mM), 10 pmol of fluorescent-labeled primers, 0.05 ml dNTP (10 mM) and 0.2 ml Taq polymerase (1 U ml 1 ) (all reagents were from Qiagen Inc., Valencia, CA, USA). PCR was performed using an MJ Research PTC-200 thermocycler. The PCR conditions were as follows: after an initial 10 min denaturation step at 95 1C, 40 amplification cycles were performed consisting of 40 s at 95 1C, 40 s at 54 1C and a 1 min elongation step at 72 1C. Amplification was completed with a final extension step at 72 1C for 7 min. The fluorescent-labeled products were finally analysed on an ABI PRISM 3100 l Genetic Analyzer (Applied Biosystems, Foster City, CA, USA). Tumors were classified as MSI if at least two or more markers out of the five were unstable and as MSS if only one or none of the markers was unstable.

### Statistical Analysis

The JMP8 (SAS Institute, Inc., Cary, NC) software package was used for data analyses. Survival curves were generated using the Kaplan-Meier method, with significance evaluated using the Mantel-Cox log-rank test. Risk ratio (relative risk for death) was calculated using the Cox Proportional Hazard model in both univariate and multivariate analyses. Comparisons between groups were made with the paired Student's t-test. Chi-square tests were used to examine relationship between nominal variables. The limit of significance for all analyses was defined as a p-value of 0.05.

## Competing interests

The authors declare that they have no competing interests.

## Authors' contributions

PB designed research, performed experiments, analyzed data, and wrote the paper; SP performed validation experiments and helped in writing the paper; JA performed experiments and analyzed data; ZQ & TG collected and analyzed data; performed statistical analysis FA provided archival pathology material, review of histopathology slides; NA, AA, LAH and SA provided fresh tissue samples, clinical data and reviewed the manuscript, SU analyzed data and helped in writing the paper; KSA designed research, analyzed data, and wrote the paper.

All authors read and approved the final manuscript.

## Supplementary Material

Additional file 1 **Additional file 1**. Table S1. Clinico-pathological characteristics and TRAIL expression in patients with colorectal carcinoma. Table S2. Cox regression analysis for overall survival of colorectal carcinoma patients: TRAIL-R1, KRAS4A and p27^kip1^. Table S3. Trail-R1 & R2 co-expression: Cox regression analysis for overall survival of patients with colorectal carcinoma. Table S4. Trail R1 in Adjuvant treated Group: Cox regression analysis for overall survival of patients with colorectal carcinoma. Table S5. Antibodies used for tissue micro array Immunohistochemical analysis. Click here for file

Additional file 2 **Additional file 2**. Prognostic significance of TRAIL-R1 & TRAIL-R2 in early and late stage CRC and Kaplan Meier survival analysis. [A] In the early Stage subgroup (I and II) CRC patients TRAIL-R1 expression was not associated with prognostic outcome (p = 0.4703). [B] In the advanced Stage subgroup(III and IV) CRC patients with over expression of TRAIL-R1(n = 157) had a better overall survival of 48.8% at 5 years as compared to 26.0% with reduced TRAIL-R1 expression (n = 30; p = 0.0060). [C] In the early Stage subgroup (I and II) CRC patients TRAIL-R2 expression was not associated with prognostic outcome (p = 0.5613). [D] In the advanced Stage subgroup(III and IV) CRC patients with over expression of TRAIL-R2 (n = 112) had a better overall survival of 50.0% at 5 years as compared to 35.2% with reduced TRAIL-R2 expression (n = 70; p = 0.0263). Click here for file

Additional file 3 **Additional file 3**. Prognostic significance of TRAIL-R1 & R2 in CRC with Stage II and III and Kaplan Meier survival analysis. [A] In the Stage subgroup(II and III) CRC patients with over expression of TRAIL-R1(n = 226) had a better overall survival of 67.6% at 5 years as compared to 63.1% with reduced TRAIL-R1 expression (n = 41; p = 0.2508). [B] In the Stage subgroup(II and III) CRC patients with over expression of TRAIL-R2(n = 149) had a better overall survival of 71.3% at 5 years as compared to 57.3% with reduced TRAIL-R2 expression (n = 105; p = 0.0088). Click here for file

Additional file 4 **Additional file 4**. Prognostic significance of co-expression of TRAIL receptors: TRAIL-R1 and TRAIL-R2 in CRC and Kaplan Meier survival analysis. The CRC subgroup with overexpression of TRAIL-R1 and TRAIL-R2 (n = 191) had a better overall survival of 69.5% at 5 years as compared to Reduced TRAIL-R1 and TRAIL-R2 expression of 57.9% (n = 145; p = 0.0107). Click here for file

Additional file 5 **Additional file 5**. Prognostic significance of TRAIL-R1 & TRAIL-R2 in CRC based on adjuvant therapy and Kaplan Meier survival analysis. [A] CRC patients with over expression of TRAIL-R1 in adjuvant treated group had a better overall survival of 70.6% at 5 years as compared to 48.4% with reduced TRAIL-R1 expression (n = 203; p = 0.0033). [B] In the non-adjuvant treated group TRAIL-R1 expression was not associated with prognostic outcome (p = 0.8801). [C] CRC patients with over expression of TRAIL-R2 in adjuvant treated had a better overall survival of 73.5% at 5 years as compared to 46.5% with reduced TRAIL-R2 expression (n = 168; p = 0.0998). [D] In the non-adjuvant treated group TRAIL-R2 expression was not associated with prognostic outcome (p = 0.8069). Click here for file
