# Supplementary material for: Prognostic significance of TRAIL death receptors in Middle Eastern colorectal carcinomas and their correlation to oncogenic KRAS alterations
Source: Mol Cancer. 2010 Jul 30;9:203. doi: 10.1186/1476-4598-9-203 (PMC2922191; doi:10.1186/1476-4598-9-203)
Supplement: Additional file 1 — Table S1. Clinico-pathological characteristics and TRAIL expression in patients with colorectal carcinoma. Table S2. Cox regression analysis for overall survival of colorectal carcinoma patients: TRAIL-R1, KRAS4A and p27kip1. Table S3. Trail-R1 & R2 co-expression: Cox regression analysis for overall survival of patients with colorectal carcinoma. Table S4. Trail R1 in Adjuvant treated Group: Cox regression analysis for overall survival of patients with colorectal carcinoma. Table S5. Antibodies used for tissue micro array Immunohistochemical analysis. [file 1476-4598-9-203-S1.DOC]

Additional File 1

Table S1. Clinico-pathological characteristics and TRAIL expression of patients with colorectal carcinoma.

|  |  | | **High TRAIL** | | **Low TRAIL** | | **P value** |
| --- | --- | --- | --- | --- | --- | --- | --- |
|  | **N** | **%** | **N** | **%** | **N** | **%** |
| **Total Number of Cases** | 403 |  | 127 | 31.5 | 276 | 68.5 |  |
| **Age** |  |  |  |  |  |  |  |
| <= 50 years | 132 | 32.7 | 38 | 28.8 | 94 | 71.2 | 0.4090 |
| >50 years | 271 | 67.3 | 89 | 32.8 | 182 | 67.2 |  |
| **Gender** |  |  |  |  |  |  |  |
| Male | 198 | 49.1 | 60 | 30.3 | 138 | 69.7 | 0.6071 |
| Female | 205 | 50.9 | 67 | 32.7 | 138 | 67.3 |  |
| **Tumour Site** |  |  |  |  |  |  |  |
| Left colon | 336 | 83.4 | 106 | 31.6 | 230 | 68.4 | 0.9738 |
| Right colon | 67 | 16.6 | 21 | 31.3 | 46 | 68.7 |  |
| **Histological Type** |  |  |  |  |  |  |  |
| Adenocarcinoma | 351 | 87.1 | 111 | 31.6 | 240 | 68.4 | 0.9013 |
| Mucinous Carcinoma | 52 | 12.9 | 16 | 30.8 | 36 | 69.2 |  |
| **Tumour Stage** |  |  |  |  |  |  |  |
| I | 58 | 15.1 | 24 | 41.4 | 34 | 58.6 | 0.3259 |
| II | 126 | 32.8 | 38 | 30.2 | 88 | 69.8 |  |
| III | 149 | 38.8 | 42 | 28.2 | 107 | 71.8 |  |
| IV | 51 | 13.3 | 17 | 33.3 | 34 | 66.7 |  |
| **Differentiation** |  |  |  |  |  |  |  |
| Well | 30 | 7.4 | 8 | 26.7 | 22 | 73.3 | 0.8000 |
| Moderate | 301 | 74.7 | 95 | 31.6 | 206 | 68.4 |  |
| Poor | 72 | 17.9 | 24 | 33.3 | 48 | 66.7 |  |
| **KRAS Mutation** |  |  |  |  |  |  |  |
| Present | 77 | 29.7 | 21 | 27.3 | 56 | 72.7 | 0.4600 |
| Absent | 182 | 70.3 | 58 | 31.9 | 124 | 68.1 |  |
| **K-RAS 2A** |  |  |  |  |  |  |  |
| High K-RAS 2A | 151 | 45.7 | 53 | 35.1 | 98 | 64.9 | 0.7632 |
| Low K-RAS 2A | 179 | 54.3 | 60 | 33.5 | 119 | 66.5 |  |
| **K-RAS 2B** |  |  |  |  |  |  |  |
| High K-RAS 2B | 110 | 33.2 | 36 | 32.7 | 74 | 67.3 | 0.7819 |
| Low K-RAS 2B | 221 | 66.8 | 69 | 31.2 | 152 | 68.8 |  |
| **Cleaved-Caspase 3** |  |  |  |  |  |  |  |
| High Cleaved-Caspase 3 | 165 | 49.6 | 65 | 39.4 | 100 | 60.6 | 0.0643 |
| Low Cleaved-Caspase 3 | 168 | 50.4 | 50 | 29.8 | 118 | 70.2 |  |
| **P27 (Nuc)** |  |  |  |  |  |  |  |
| High P27 | 141 | 38.8 | 48 | 34.0 | 93 | 66.0 | 0.4417 |
| Low P27 | 222 | 61.2 | 67 | 30.2 | 155 | 69.8 |  |
| **MSI-Molecular** |  |  |  |  |  |  |  |
| MSI-H | 71 | 19.3 | 24 | 33.8 | 47 | 66.2 | 0.6074 |
| MSI-S/L | 297 | 80.7 | 91 | 30.6 | 206 | 69.4 |  |
| **Overall Survival** |  |  |  |  |  |  | 0.2901 |
| 5 Years |  |  |  | 59.9 |  | 68.1 |  |

Table S2 Cox regression analysis for overall survival of colorectal carcinoma patients: TRAIL-R1, KRAS4A and p27^kip1^.

| **Clinical Parameters** | **UNIVARIATE** | | **MULTIVARIATE** | |
| --- | --- | --- | --- | --- |
|  | Risk Ratio  (95% CI) | p value | Risk Ratio  (95% CI) | p value |
| Age: Above=50 | 1.17 (0.80-1.74) | 0.4306 | 1.00 (0.58-1.76) | 0.9913 |
| Sex: Male | 1.10 (0.76-1.59) | 0.6084 | 1.00 (0.60-1.65) | 0.9981 |
| Stage: III-IV | 7.26 (4.34-13.03) | <0.0001 | 8.22 (4.16-18.66) | < 0.0001 |
| Grade: poorly differentiated | 1.41 (0.90-2.14) | 0.1307 | 1.49 (0.77-2.73) | 0.2219 |
| MSI status: MSI-L/S | 2.04 (1.14 – 4.05) | 0.0149 | 3.00 (1.41-7.42) | 0.0030 |
| p27^kip1^: Low | 2.07 (1.32-3.39) | 0.0012 | 1.73 (1.00-3.12) | 0.0498 |
| KRAS4A:Low | 1.86 (1.21-2.95) | 0.0046 | 1.85 (1.06-3.28) | 0.0288 |
| TRAIL-R1:Low expression | 1.80 (1.11-2.83) | 0.0196 | 1.80 (0.91-3.31) | 0.0883 |

CI = confidence interval

Table S3**.** Trail-R1 & R2 co-expression: Cox regression analysis for overall survival of patients with colorectal carcinoma.

| **Clinical Parameters** | **UNIVARIATE** | | **MULTIVARIATE** | |
| --- | --- | --- | --- | --- |
|  | Risk Ratio  (95% CI) | p value | Risk Ratio  (95% CI) | p value |
| **Age:** Above=50 | 1.17 (0.80-1.74) | 0.4306 | 1.41 (0.83-2.44) | 0.1957 |
| **Sex:** Male | 1.10 (0.76-1.59) | 0.6084 | 1.21 (0.74-1.94) | 0.4520 |
| **Stage:** III-IV | 7.26 (4.34-13.03) | **<0.0001** | 6.14 (3.35-12.36) | <0.0001 |
| **Grade:** Poorly differentiated | 1.41 (0.90-2.14) | 0.1307 | 1.54 (0.81-2.79) | 0.1799 |
| **MSI status:** MSI-L/S | 2.04 (1.14 – 4.05) | **0.0149** | 2.37 (1.14-5.80) | 0.0187 |
| **TRAIL-R1& R2:** Low expression | 1.72 (1.13-2.64) | 0.0112 | 1.71 (1.04-2.79) | 0.0353 |

CI = confidence interval

Table S4. Trail R1 in Adjuvant treated Group: Cox regression analysis for overall survival of patients with colorectal carcinoma.

| **Clinical Parameters** | **UNIVARIATE** | | **MULTIVARIATE** | |
| --- | --- | --- | --- | --- |
|  | Risk Ratio (95% CI) | p value | Risk Ratio (95% CI) | p value |
| **Age:** Above=50 | 1.17 (0.80-1.74) | 0.4306 | 1.62 (0.85-3.21) | 0.1433 |
| **Sex:** Male | 1.10 (0.76-1.59) | 0.6084 | 0.86 (0.46-1.58) | 0.6341 |
| **Stage:** III-IV | 7.26 (4.34-13.03) | **<0.0001** | 7.63 (3.04-25.64) | **<0.0001** |
| **Grade:** Poorly differentiated | 1.41 (0.90-2.14) | 0.1307 | 2.51 (1.26-4.83) | 0.0094 |
| **MSI status:** MSI-L/S | 2.04 (1.14 – 4.05) | **0.0149** | 1.51 (0.70-3.77) | 0.3076 |
| **TRAIL-R1:** Low expression | 1.80 (1.11-2.83) | **0.0196** | 2.17 (1.08-4.16) | 0.0306 |

CI = confidence interval

###### Table SS5. Antibodies used for tissue micro array Immunohistochemical analysis.

| **Antibody** | **Positive cases (%)** | **Catal**  **ogue** | **Clone** | **Company** | **Source** | **Dilution** | **Antigen**  **Retrieval** |
| --- | --- | --- | --- | --- | --- | --- | --- |
| **(Sub cellular Localization)** |  |  |  |  |  | **#** |  |
| Trail mAb | 127/403  (31.5%) | #-3219 | C92B9 | Cell signaling Technology | rabbit | 1:200 | pH9 |
| Anti Trail R1 | 331/387  (85.5%) | AF-347 | Polyclonal | R&D systems | goat | 1:500 | pH6 |
| Anti Trail R2 | 217/365  (59.4%) | AF-631 | Polyclonal | R&D systems | goat | 1:1000 | pH6 |
| KRAS2A | 161/353  (45.6%) | Sc-522 | Polyclonal | SCBT | rabbit | 1:500 | pH9 |
| KRAS2B | 123/371  (33.1%) | Sc-521 | Polyclonal | SCBT | rabbit | 1:500 | pH9 |
| C-Casp3 (ASP175-5A1) | 175/356  (49.2%) | # - 9664 | ASP-175  (5aIE) | CST | rabbit | 1:500 | pH6 |
| p27 | 150/393  (38.2%) | 18-2370 | 57 | Zymed | mouse | 1:300 | pH9 |

**#** overnight incubation
