# Supplementary figures and images for: Prognostic significance of TRAIL death receptors in Middle Eastern colorectal carcinomas and their correlation to oncogenic KRAS alterations
Source: Mol Cancer. 2010 Jul 30;9:203. doi: 10.1186/1476-4598-9-203 (PMC2922191; doi:10.1186/1476-4598-9-203)

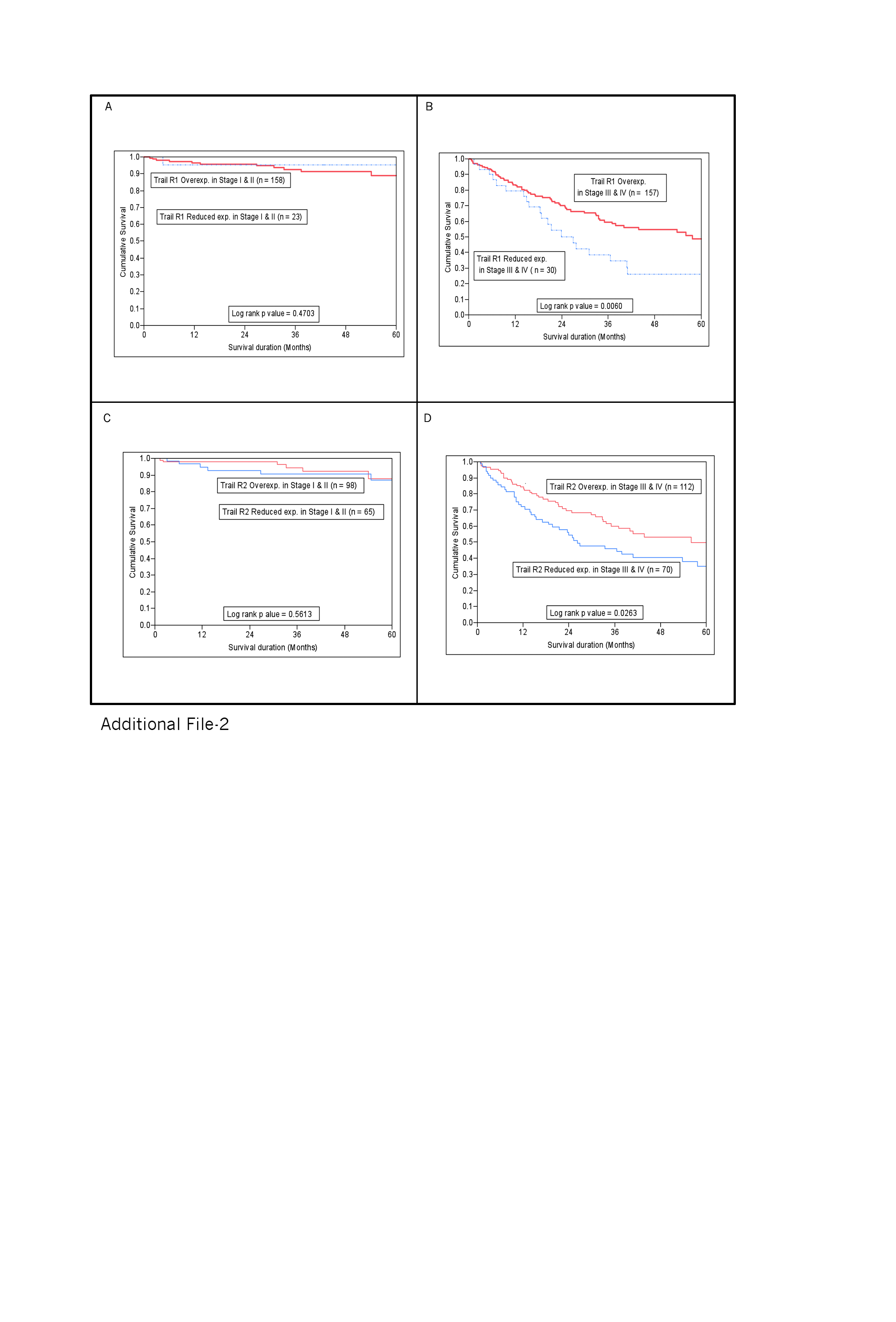

Supplement: Additional file 2 — Prognostic significance of TRAIL-R1 & TRAIL-R2 in early and late stage CRC and Kaplan Meier survival analysis. [A] In the early Stage subgroup (I and II) CRC patients TRAIL-R1 expression was not associated with prognostic outcome (p = 0.4703). [B] In the advanced Stage subgroup(III and IV) CRC patients with over expression of TRAIL-R1(n = 157) had a better overall survival of 48.8% at 5 years as compared to 26.0% with reduced TRAIL-R1 expression (n = 30; p = 0.0060). [C] In the early Stage subgroup (I and II) CRC patients TRAIL-R2 expression was not associated with prognostic outcome (p = 0.5613). [D] In the advanced Stage subgroup(III and IV) CRC patients with over expression of TRAIL-R2 (n = 112) had a better overall survival of 50.0% at 5 years as compared to 35.2% with reduced TRAIL-R2 expression (n = 70; p = 0.0263). [file 1476-4598-9-203-S2.TIFF]

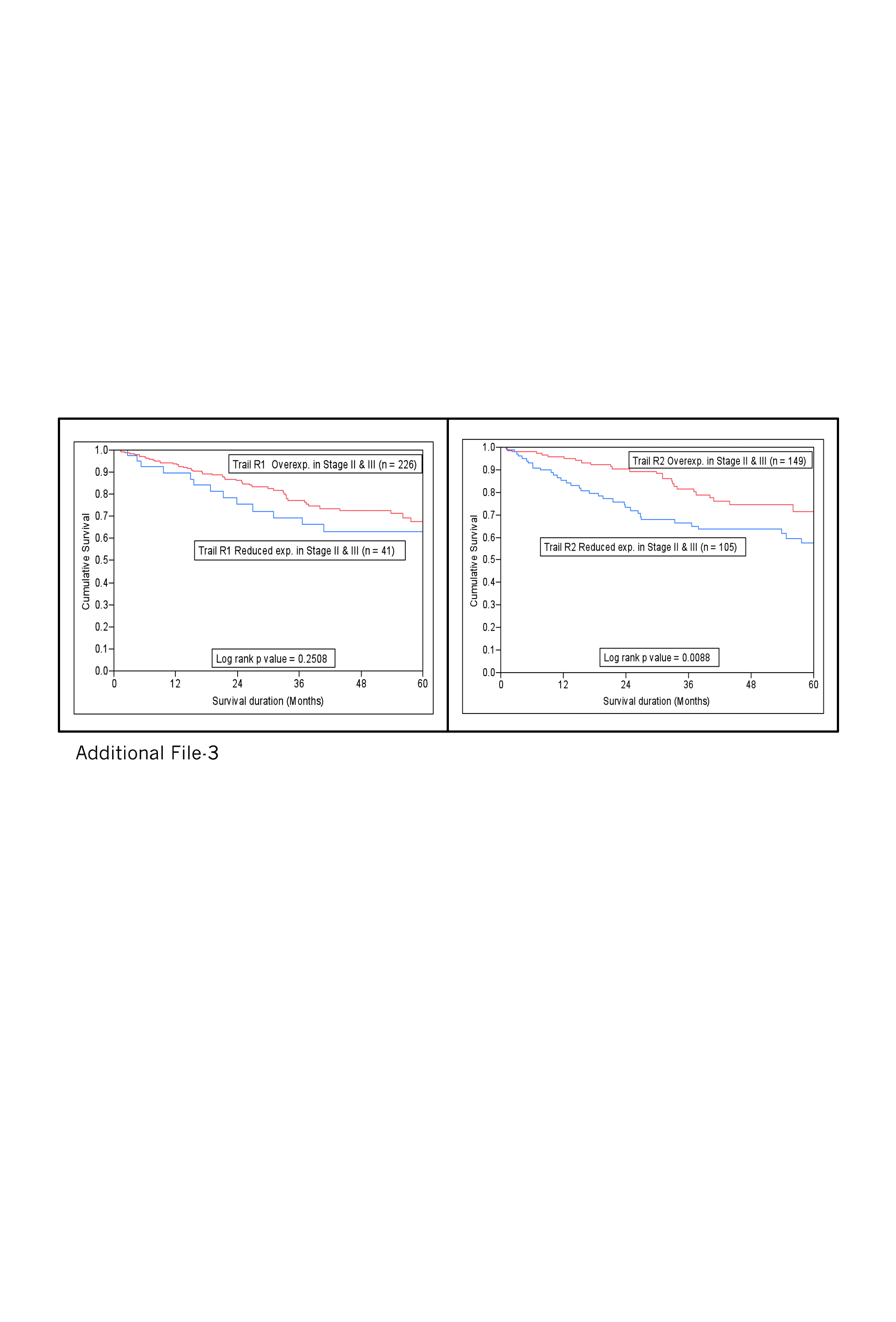

Supplement: Additional file 3 — Prognostic significance of TRAIL-R1 & R2 in CRC with Stage II and III and Kaplan Meier survival analysis. [A] In the Stage subgroup(II and III) CRC patients with over expression of TRAIL-R1(n = 226) had a better overall survival of 67.6% at 5 years as compared to 63.1% with reduced TRAIL-R1 expression (n = 41; p = 0.2508). [B] In the Stage subgroup(II and III) CRC patients with over expression of TRAIL-R2(n = 149) had a better overall survival of 71.3% at 5 years as compared to 57.3% with reduced TRAIL-R2 expression (n = 105; p = 0.0088). [file 1476-4598-9-203-S3.TIFF]

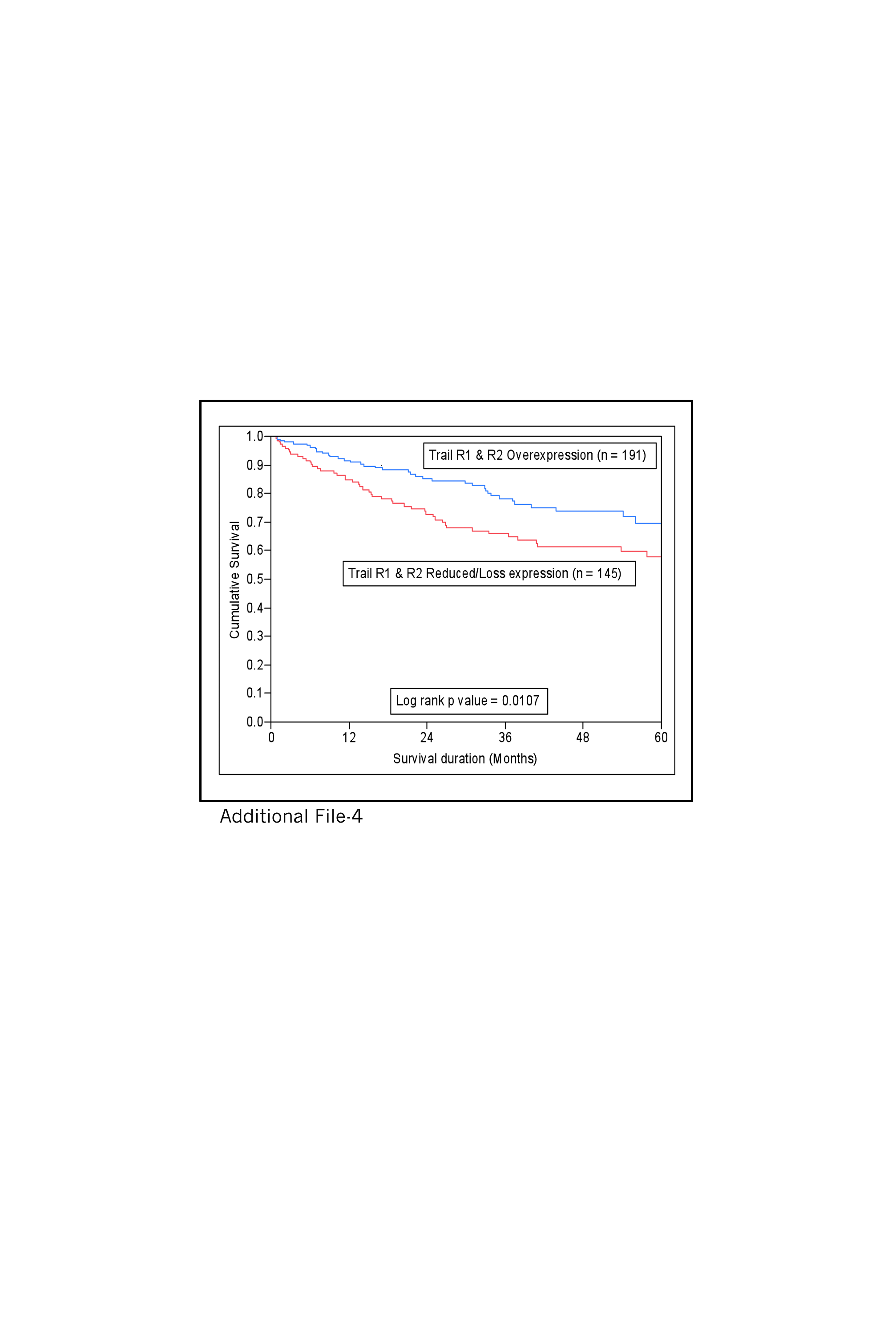

Supplement: Additional file 4 — Prognostic significance of co-expression of TRAIL receptors: TRAIL-R1 and TRAIL-R2 in CRC and Kaplan Meier survival analysis. The CRC subgroup with overexpression of TRAIL-R1 and TRAIL-R2 (n = 191) had a better overall survival of 69.5% at 5 years as compared to Reduced TRAIL-R1 and TRAIL-R2 expression of 57.9% (n = 145; p = 0.0107). [file 1476-4598-9-203-S4.TIFF]

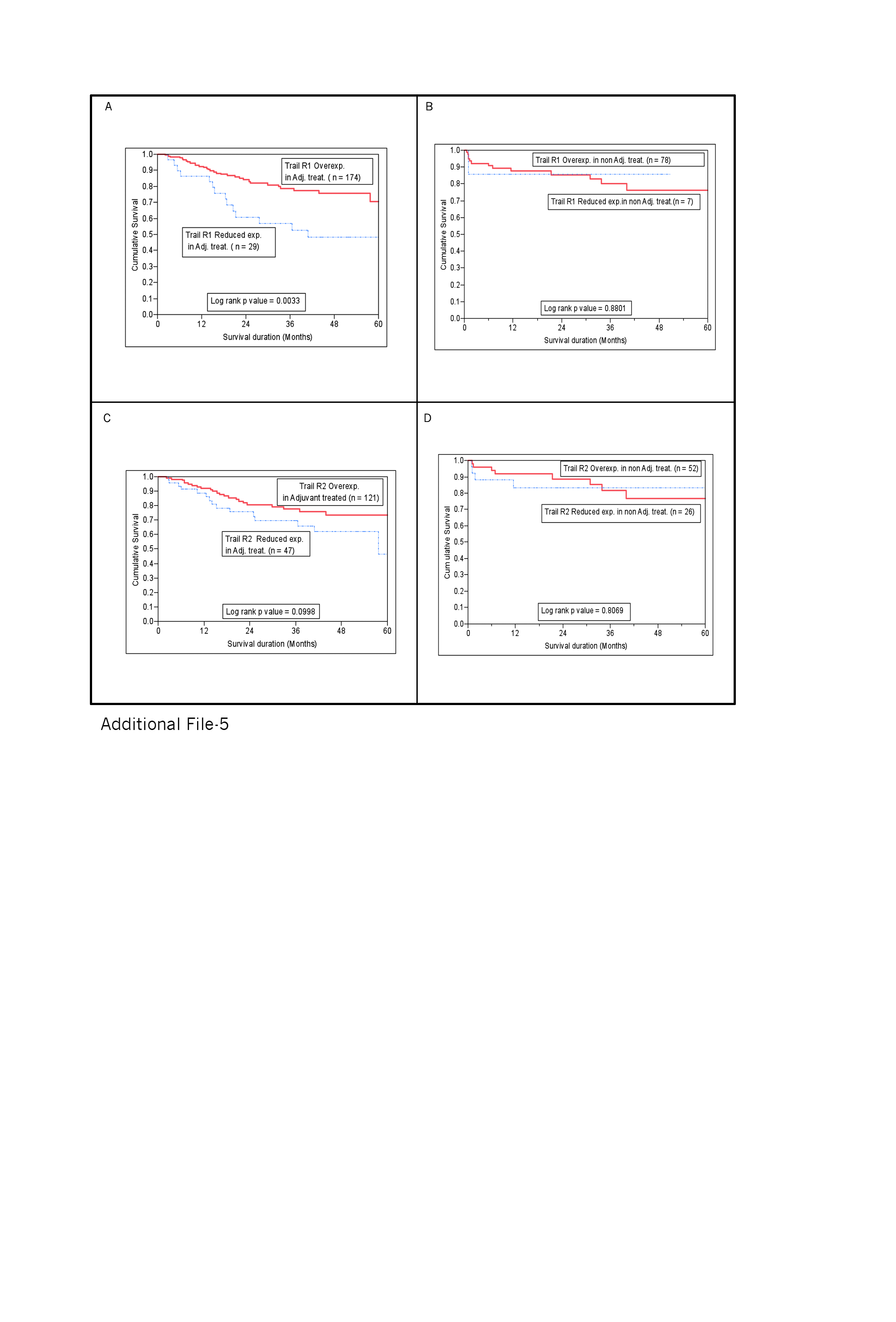

Supplement: Additional file 5 — Prognostic significance of TRAIL-R1 & TRAIL-R2 in CRC based on adjuvant therapy and Kaplan Meier survival analysis. [A] CRC patients with over expression of TRAIL-R1 in adjuvant treated group had a better overall survival of 70.6% at 5 years as compared to 48.4% with reduced TRAIL-R1 expression (n = 203; p = 0.0033). [B] In the non-adjuvant treated group TRAIL-R1 expression was not associated with prognostic outcome (p = 0.8801). [C] CRC patients with over expression of TRAIL-R2 in adjuvant treated had a better overall survival of 73.5% at 5 years as compared to 46.5% with reduced TRAIL-R2 expression (n = 168; p = 0.0998). [D] In the non-adjuvant treated group TRAIL-R2 expression was not associated with prognostic outcome (p = 0.8069). [file 1476-4598-9-203-S5.TIFF]
